# Measuring adolescent health literacy in Taiwan: validation of the health literacy assessment scale for adolescents

**DOI:** 10.1186/s12889-023-17167-5

**Published:** 2023-12-04

**Authors:** An-Kuo Chou, Chun-Hua Liao, Duan-Rung Chen

**Affiliations:** 1 Department of Pediatrics, National Taiwan University Hospital Hsin-Chu Branch, 30059 Hsinchu, Taiwan; 2 Institute of Health Policy and Management, College of Public Health, National Taiwan University, 10055 Taipei, Taiwan; 3 Population Health Research Center, National Taiwan University, 10055 Taipei, Taiwan; 4 Institute of Health Behaviors and Community Sciences, College of Public Health, National Taiwan University, No. 17, Xu-Zhou Rd., Taipei, 10055 Taiwan

**Keywords:** Health literacy, Psychometric properties, Adolescent, Health literacy inequality

## Abstract

**Background:**

This study aimed to validate the Chinese version of the Health Literacy Assessment Scale for Adolescents (HAS-A) and conduct a comparative analysis of adolescent health literacy between Taiwan and other countries.

**Methods:**

The Chinese version of the HAS-A was completed by 2,312 adolescents in the fifth and sixth grades of a primary school. Psychometric properties were examined using consistent internal reliability and confirmatory factor analysis. These assessments were compared with the results from different regions to explore health literacy inequality.

**Results:**

Construct validity was good, and internal consistency was acceptable. The scale, particularly regarding communication health literacy, was associated with parents’ socioeconomic status, and family income had a more significant impact on children’s health literacy than community income. Health literacy disparities appear in different countries, with Taiwan exhibiting the lowest level of communication health literacy.

**Conclusion:**

The results indicate that the HAS-A is a valuable tool for assessing the health literacy of 10–11-year-old adolescents and can uncover health literacy inequality among different regions.

**Supplementary Information:**

The online version contains supplementary material available at 10.1186/s12889-023-17167-5.

## Background

Enhancing health literacy is a primary strategy for improving health behaviors and disease outcomes [1]. Health literacy comprises the skills to obtain, understand, appraise, and apply health information in various life situations for healthcare, disease prevention, and health promotion [2–4]. Health literacy levels determine how individuals use their skills [5]. The fundamental functional health literacy refers to an individual’s ability to read, write, calculate, understand, and use health information. Interactive health literacy refers to an individual’s ability to interact with healthcare providers and use the information received to manage their health, including communication, coordination, and decision-making skills [3, 5]. Critical health literacy refers to an individual’s ability to analyze and evaluate health information from various sources, such as the media or healthcare providers [5].

Numerous studies emphasize the pivotal role health literacy plays in determining health outcomes. For adults and the elderly, lower health literacy has been linked to more frequent visits to the emergency department [6, 7], an increased utilization of healthcare services [8], elevated hospitalization and mortality rates among patients with chronic conditions [9–11]. Interventions targeting those with lower health literacy have improved in health outcomes [12]. In the context of adolescents, there’s emerging evidence to suggest significant associations between health literacy and health-promoting behaviors [13, 14], substance abuse patterns such as alcohol and smoking [15, 16], and overall health outcome [17, 18]. Nevertheless, while abundant research has been conducted on adults, investigations into adolescent health literacy are notably sparse, with much of the current literature leaning toward theoretical discussions [19, 20]. Appropriately assessing children’s health literacy can be challenging. First, their cognitive, thinking, language, memory, and comprehension abilities constantly change with age and developmental stages. Therefore, measuring health literacy in different age groups of children requires using different questions and assessment tools to ensure the validity and reliability of the measurement [21]. Second, children’s health knowledge and behaviors may need to be consistent. Children may have higher levels of health knowledge, but this may not translate into health behaviors [22]. Third, owing to limited language expression, inadequate vocabulary, poor comprehension, and a lack of experience, children may provide ambiguous, uncertain, or misunderstood responses [23]. Less health-related knowledge and experience in the healthcare system and less autonomy in making health decisions may result in biased answers when measuring children’s health literacy.

Health literacy is the ability to build a personal and community-based understanding of health [24]. It is developed over time and is related to their accumulation of experience using external health information [25] and affected by family characteristics, including parent education and income [26, 27]. In this process, children and adolescents become increasingly responsible for their health and managing various health-related issues, forming their views on health issues and developing skills that can impact their health and well-being. Therefore, having well-validated instruments to measure children’s health literacy and developing appropriate assistive tools for those with lower health literacy will assist children and adolescents manage their health sustainably. However, a well-validated instrument needs to be improved to assess children’s health literacy in Taiwan and compare it with those in other regions.

The Health Literacy Assessment Scale for Adolescents (HAS-A) is a well-developed multidimensional instrument for assessing health literacy in adolescents [28]. HAS-A has 15 items related to three dimensions (“Communication: communicating health information,” “Confusion: confusion about health information,” and “Functional: understanding health information”). The HAS-A was designed to be self-administered by adolescents in general settings, starting from the age of 10, and has been translated into several languages with strong validation [28–30].

To promote health literacy as a critical factor in prevention and health promotion among children in the general population, this study aimed to translate the HAS-A into Chinese and investigate the psychometric properties of three health literacy assessment tools. Additionally, this study aimed to compare the health literacy subscales between Taiwan and other countries using the HAS-A.

## Methods

### Participants

The target group consisted of fifth- and sixth-grade children attending elementary schools in Hsinchu County, Taiwan. Data were collected only from these children. We chose this age group because children at this stage, early adolescent, are expected to possess the necessary language skills for a written survey, develop abstract thinking skills, comprehend the complexity of causal relationships, and obtain knowledge from their experiences and observations.

In our study design, we anticipated an effect size of 0.1 and aimed for a statistical power level of 0.8. Given that there were 3 original latent variables and 15 observed items, with a probability level set at 0.01, we referred to the a-priori sample size calculator [31–33]. This indicated that a minimum of 1719 samples was required to detect the desired effect.

The sampling method for study participants was as follows: In the first stage, at the end of August 2020, the population size of fifth- and sixth-grade students (aged 10–11 years according to Taiwan’s primary school admission criteria) in each township of Hsinchu County was collected and allocated according to the population proportion of each township. In the second stage, the probability proportional to size sampling was used to select schools in each township. All the schools selected in the second stage were evenly divided into fifth and sixth grades based on the number of classes assigned to each school, and the classes were randomly selected. In the final stage, all students in each selected class were invited to participate in the study. Using the above sampling strategy, 3,620 adolescents from 30 schools were selected for the formal survey, representing 15.23% of the 10–11-year-olds in Hsinchu County.

Before commencing the study, we adopted a two-step approach. Initially, students were briefed face-to-face in their classrooms regarding the research’s aims and methodology. Concurrently, a detailed research plan and a questionnaire were provided for students to disseminate to their parents. Recognizing the survey’s anonymous design, participants were free to abstain from submitting or returning a blank form. Our research plan offered explicit contact avenues, inviting any questions or concerns from both students and parents. To ensure personal privacy and protect sensitive populations, our study used an anonymous, one-time questionnaire, and eliminating the need to retain participants’ names that did not involve direct contact with human participants. Therefore, the Research Ethics Committee at National Taiwan University Hospital Hsin-Chu Branch in Hsinchu, Taiwan, approved the waiver of written informed consent for this study. Participants who chose not to participate were provided with the option to return a sealed envelope containing a blank questionnaire. This would prevent individuals from being identified in the class. Phonetic symbols were added to the questionnaire to make the items more explicit. All the questionnaires were completed at home.

This study was conducted at the National Taiwan University Hospital Hsin-Chu Branch. It was initiated after obtaining approval from the Research Ethics Committee, National Taiwan University Hospital Hsin-Chu Branch, Hsinchu, Taiwan (Institutional Review Board tracking number: 109-145 F).

### Measurements

To help validate the Chinese translation of HAS-A, we employed one Chinese translator to translate the original English version of the questionnaire into Chinese. Three pediatric health and public health scholars reviewed and modified the questionnaire, including two pediatric specialists and one public health professor. Finally, the translation process was checked, and the opinions of three adolescents, aged 10–11 years, were considered before the final Chinese version of the questionnaire was compiled.

The questionnaire’s response categories were phrased similarly: “On a scale from never to always, how often does your doctor understand what you mean when you ask [them] a question about your health?” They were rated on a five-point Likert-type scale (0 = never, 1 = rarely, 2 = sometimes, 3 = usually, 4 = always), and items 6–15 were reverse-coded. The original health literacy questionnaire has three subscales: the communication scale (communicating health information), the confusion scale (confusion about health information), and the functional scale (understanding health information). The original scale defines a score of less than 15 on the communication scale as indicating low health literacy. Additionally, scores of 8 or higher on the confusion scale and 12 or higher on the functional scale indicate low health literacy.

The following demographic and socioeconomic characteristics were analyzed: sex (male and female), school grade (fifth and sixth grade), educational level of father and mother (at least one parent ≤ high school and below, both parents ≥ college/university and above), monthly family income in ten thousand New Taiwan Dollars (NTD) (< 3, 3–10, > 10), and town scale (higher income (above the median), lower income (below the median), based on the 2020 National Household Income Statistics) [34].

### Statistical analysis

Internal consistency was examined using Cronbach’s alpha and McDonald’s omega. The distributional properties of the instrument were further examined to determine the normality of the scores on each subscale and identify floor and ceiling effects. Floor or ceiling effects were considered present if > 15% of the patients scored the lowest or highest possible score at the subscale level [35]. At the item level, these effects were present if more than 75% of respondents answered in the lowest or highest response category [36].

Confirmatory factor analysis (CFA) was used to assess the construct validity of the HAS-A. The adjusted goodness-of-fit index (AGFI), root mean square error of approximation (RMSEA), comparative fit index (CFI), and normed fit index (NFI) were used as model fit indices in CFA. An AGFI value of 0.90 or greater is generally considered to indicate an acceptable model fit. An RMSEA value of less than 0.05 represents a good fit, and a value of less than 0.08 is acceptable. CFI and NFI values of 0.90 or greater are generally considered to indicate an acceptable model fit [37].

Some scales were used to confirm the criterion validity. The first was the Sugar-Sweetened Beverage Self-Efficacy Scale (SSB-SE), comprising 10 items [38]. The self-efficacy scale consists of three subscales: mental exhaustion, need for company, and high accessibility. Participants rated their confidence in controlling their sugar-sweetened beverage intake on a scale from 0 (not sure at all) to 3 (extremely sure). The higher the score, the more confident the adolescents were in controlling their sugar-sweetened beverage intake. Emotional eating behavior was assessed using the Chinese version of the Emotional Eating Scale (EES) extracted from the Three-Factor Eating Questionnaire [39]. This scale includes three items measured on a scale from 1 (definitely wrong) to 4 (definitely suitable) to assess children’s emotional eating behavior. The higher the score, the more pronounced the children’s emotional eating behavior. Convergent validity was assessed by examining average variance extracted (AVE) and composite reliability (CR) values. An AVE value of 0.360 or greater [40] and a CR value of 0.500 or greater [41] indicate acceptable convergent validity. We also utilized two self-reported health behavior measures from adolescents. The first metric pertains to the duration of outdoor activities. This represents the average daily time students spend engaged in outdoor activities, such as walking, playground activities, ball games, or other forms of exercise. We categorized the duration into intervals: less than 30 min, 31–60 min, 61–90 min, and over 90 min. The second metric focuses on screen time, indicating the average daily duration students spend watching television, playing video games, or engaging in other screen-related activities. This was similarly categorized into intervals: less than 30 min, 31–60 min, 61–90 min, and more than 90 min.

The HAS-A has been translated into other languages and used to measure adolescent health literacy [28–30]. Therefore, our results will be compared with the results of other surveys to explore differences in adolescent health literacy across regions.

To explore the factors associated with adolescent`s health literacy, we used Cohen’s d to calculate effect sizes (corrected for variable group sizes, if necessary). The effect sizes were small if d = 0.2, medium if d = 0.5, and significant if d = 0.8. We compared the mean scores for each health literacy scale using Student’s t-tests between adolescents according to several variables: sex (no difference), school grade (higher health literacy for higher school grade), a parental education level (higher health literacy for higher education level), family monthly income (higher health literacy for higher family income), and town scale (higher health literacy if living in a high-income town).

Statistical tests were two-tailed, and a p-value < 0.05 was considered significant. All analyses were performed using IBM SPSS Statistics for Windows, version 27.0. We used CFA with IBM Amos 24.0 to assess the structural validity of the measurement model.

## Results

In this study, 63.9% (n = 2,312) of the students agreed to participate. The sex and school grade ratios were balanced. Regarding parental education, 66.1% had attained a college or university degree or higher. Family income was within 3–10 thousand NTD for more than half of the participants (50.9%). Of all the participants, 53.2% lived in high-income townships (Table 1).

**Table 1:** Characteristics of Study Participants (n = 2,312)

	n	%
Sex		
Male	1,192	51.6
Female	1,120	48.4
Grade		
5th grade	1,208	52.2
6th grade	1,104	47.8
Parents’ education (n = 1948)		
At least one parent ≤ high school and below	661	33.9
Both parents ≥ college/university and above	1,287	66.1
The monthly family income (ten thousand NTD*) (n = 2,228)		
< 3	128	5.5
3–10	1,133	49.0
> 10	967	41.8
Town scale **		
High income (above the median)	1,230	53.2%
Low income (below the media)	1,082	46.8%

*$1 = about 30 NTD in April 2023

** Based on the 2020 national household income [34]

The distribution of responses for each item of the HAS-A is shown in Supplementary Table 1. At the subscale level, the communication scale had a prevalence of 9.2% for the highest score. In contrast, the confusion and functional scales had proportions of 12.8% and 11.6% for the lowest scores, respectively. The results of our study did not show ceiling or floor effects on these subscales.

The internal consistency of the four health literacy indices was assessed in terms of reliability. The Cronbach’s alpha for the three subscales ranged from 0.604 to 0.841, all surpassing the 0.600 benchmark. The overall scale, comprising 15 items, exhibited a Cronbach’s alpha of 0.786, denoting satisfactory internal consistency. McDonald’s omega coefficient was 0.705 for the overall health literacy scale and 0.842, 0.623, and 0.766 for the communication, confusion, and functional subscales, respectively (Table 2).

**Table 2:** Reliability and Criterion Validity of Health Literacy Indices

Subscale of HAS-A	Cronbach’s alpha	McDonald’s omega	Spearman’s correlation coefficients			
			SSB-SE	EES	Outdoor activity time	Screen time
Communication	0.841	0.842	0.221**	-0.172**	0.152**	-0.073**
Confusion *	0.604	0.623	-0.127**	0.180**	-0.031	0.027
Functional *	0.763	0.766	-0.174**	0.201**	-0.096**	0.040

HAS-A: Health Literacy Assessment Scale for Adolescents; SSB-SE: the Sugar-Sweetened Beverage Self-Efficacy Scale; EES: the Emotional Eating Scale; the higher the score, the more prominent the emotional eating behavior

* Higher scores mean lower health literacy

** p < .001

Inter-item correlations for all items of the communication scale were > 0.3, whereas for the confusion scale, the inter-item correlations between items Q6 and Q7, Q8, and Q9 were slightly below 0.3. Similarly, on the functional scale, the inter-item correlation between items Q15, Q10, and Q12 was slightly below 0.3 (Supplementary Table 2). The average inter-item correlations for the subscales ranged from 0.300 to 0.502, and the overall average inter-item correlation was 0.398. The criterion validity of the HAS-A was assessed by examining its bivariate relationships with the SSB-SE and EES, as shown in Table2. The results indicated that all HAS-A subscales were significantly associated with the SSB-SE and EES.

Construct validity was analyzed using CFA, which revealed factor loadings of items ranging from 0.327 to 0.825, with only Q6 items below 0.400. The composite reliability of each domain was 0.632–0.848, and the AVE of the communication domain was above 0.500, whereas that of the others was only slightly below 0.360 (Supplementary Table 3). We tested three models: a single-dimensional model (model 1), a three-dimensional model excluding item Q6 (model 2), and the original three-dimensional model (model 3). Table3 shows the fit indices for all models, with Model 3 performing best on all model fit indices and the values for the CFI indicating excellent model fit (≥ 0.95) (Table 3).

**Table 3:** Construct Validity of the Chinese Version of HAS-A with Goodness-Of-Fit Indices

Model	Item	Dimension	AGFI	RMSEA	NFI	CFI
1	1–15	only one	0.553	0.150	0.510	0.514
2	1–5 7–9 10–15	communication confusion functional	0.943	0.057	0.923	0.931
3	1–5 6–9 10–15	communication confusion functional	0.955	0.049	0.942	0.950

* AGFI = adjusted goodness of fit index; CFI = comparative fit index; NFI = normed fit index; RMSEA = root mean square error of approximation. Comparing Models 2 and 3, the difference in NFI was 0.019 and the p-value was < 0.001

According to the results presented in Table 4, the communication, confusion, and functional subscales of health literacy were analyzed in different countries. The results showed that on the communication scale, the USA had the highest score (mean = 15) and a high proportion of individuals with high communication health literacy. Other countries, including Taiwan, Palestine, and France, had similar mean scores. Contrastingly, Taiwan had the highest proportion of individuals with high health literacy on the confusion and functional scales.

**Table 4:** Comparison of the Health Literacy Subscale Between Taiwan and Other Countries

Health literacy subscale		Taiwan (n = 2,312)	USA (n = 272) [21]	Palestine (n = 1,200) [22]	France (n = 1,444) [23]
	Age (years)	10–11	12–19	11–16	13–17
Communication	Mean (SD)	13.1 (4.7)	15.0	13.0 (5.3)	13.4 (3.4)
	Median	13	15	14	-
	High health literacy (%)	41.9	62.6	44.9	-
	Low health literacy (%)	58.1	37.4	55.1	-
Confusion	Mean (SD)	4.1 (3.1)	-	5.4 (3.8)	4.6 (3.2)
	Median	4	5	5	-
	High health literacy (%)	85.8	78.7	68.8	-
	Low health literacy (%)	14.2	21.3	31.2	-
Functional	Mean (SD)	5.9 (4.4)	-	7.0 (4.9)	7.0 (4.5)
	Median	5	8	6	-
	High health literacy (%)	88.7	82.4	80.3	-
	Low health literacy (%)	11.3	17.6	19.7	-

High health literacy subscale scores: communication (15–20), confusion (0–7), and functional (0–11)

As shown in Table 5, the communication subscale of the HAS-A associated with child school grade, parent’s education, and family income has a small effect size according to Cohen’s d (except for sex and town scale).

**Table 5:** Factor Possibly Associated With the Health Literacy Assessment Scale for Adolescents (HAS-A), Mean ± SD

	Communication	Confusion*	Functional*
Sex: Cohen d (*p*-value)	0.08 (0.062)	0.01 (0.755)	0.001 (0.975)
Boys	13.24 ± 4.76	4.09 ± 3.15	5.87 ± 4.52
Girls	12.87 ± 4.70	4.13 ± 3.07	5.87 ± 4.20
School Grade: Cohen d (*p*-value)	0.13 (0.002)	0.08 (0.511)	0.13(0.003)
5th grade	12.77 ± 4.70	4.23 ± 3.23	6.13 ± 4.49
6th grade	13.38 ± 4.74	3.97 ± 2.95	5.58 ± 4.22
Parent`s education: Cohen d (*p*-value)	0.17 (< 0.001)	0.06 (0.233)	0.09 (0.852)
At least one ≤ high school	12.57 ± 4.92	4.22 ± 3.16	5.89 ± 4.46
Both parents ≥ college/university	13.37 ± 4.56	4.05 ± 3.03	5.85 ± 4.18
Family income: most significant Cohen d (*p*-value)	0.32^a^ (0.003)	0.05^b^ (0.238)	0.03^b^ (0.502)
Below 3 thousand NTD	11.88 ± 5.36	4.27 ± 3.33	6.00 ± 4.81
3–10 thousand NTD	12.92 ± 4.81	4.02 ± 3.10	5.79 ± 4.34
above 10 thousand NTD	13.36 ± 4.54	4.18 ± 3.10	5.92 ± 4.31
Town scale: Cohen d (*p*-value)	0.05 (0.230)	0.04 (0.410)	0.06 (0.156)
High income	13.17 ± 4.80	4.06 ± 3.09	5.75 ± 4.32
Low income	12.94 ± 4.65	4.16 ± 3.12	6.01 ± 4.42

Statistical tests were performed using Student’s t-test. Cohen’s d was corrected for varying group sizes if necessary. * Higher scores indicate lower health literacy. **Adapted to sex and age, according to Taiwan child body status criteria. Most significant Cohen’s d between ^a^ “below 3 thousand” and “above 10 thousand NTD,” ^b^ “below 3 thousand” and “3–10 thousand NTD.

## Discussion

This study is the first to validate the psychometric properties of the Chinese version of the HAS-A and to compare health literacy levels across countries. Our study design allowed us to draw several important conclusions. First, the Chinese version of the HAS-A was valid and reliable for measuring health literacy in adolescents, with good psychometric properties in late primary school-age children. Second, the HAS-A scale was broadly consistent with the exploratory nature of the research, particularly concerning communication health literacy, including school grade, parental education, and family income. Third, we observed regional differences in health literacy levels across countries using the HAS-A.

In our research approach, we chose a translated scale for several strategic reasons. Adolescent health literacy studies in Taiwan are notably limited. The primary tools identified include the Chinese adaptation of the short-form Test of Functional Health Literacy in Adolescents from the adult version of short-TOFHLA [42], which predominantly focuses on reading, sidelining comprehensive health literacy aspects. Additionally, the Taiwan Child Health Literacy test, grounded in Taiwan’s National Health Education Curriculum [43], necessitates around an hour with computer assistance, making it impractical for regions with limited resources, which often suffer from low health literacy levels. This highlighted the imperative need for a brief, encompassing scale tailored for Taiwanese adolescents. Further advantages in selecting an already established scale encompass saving development time, enabling international comparative studies, and leveraging the original scale’s rigorously evaluated reliability and validity, ensuring its relevance in the Taiwanese setting.

The Chinese version of the HAS-A showed good internal consistency and construct validity, as indicated by the goodness-of-fit indices. However, item Q6 had a lower factor loading value, a pattern also found in the Arabic version of the HAS-A. In developing the original HAS-A, Q6 was the only health literacy process domain item classified as a confusion subscale in the final version. This item may measure unique aspects of the construct that other items in the questionnaire do not capture. Therefore, its removal may result in a loss of information and poor model fit. Although defined differently, health literacy has generally been described as an individual-based construct with a multidimensional nature, including obtaining, processing, understanding, and communicating health-related information [3, 44]. Dealing with health-related information is conceptually similar to critical thinking and evaluating information. It refers to the skills children and adolescents need to acquire when confronted with a large amount of fragmented health information [45]. Retaining item Q6 can thus contribute to a more comprehensive assessment of adolescent’s health literacy using the HAS-A.

The study explored factors associated with health literacy and found that family socio-economic status and adolescent`s school grade was significantly associated with health literacy. However, for the community income scale, only a trend was observed, suggesting a possible association but not reaching statistical significance. While both family income and community income levels are recognized as significant determinants of health literacy [46], our findings suggest that the effect size of family income on adolescent health literacy is more pronounced than that of community income. Younger children depend more on their families for economic and social support, meaning family factors significantly influence their health literacy, health behaviors, and health outcomes [47]. This finding is consistent with the ecological model [48, 49], in which the family, as the microsystem closest to the child, is a critical factor influencing individuals.

Our study revealed disparities in the distribution of health literacy across regions. Inequalities in health literacy may be caused by health policies [50], healthcare systems [51], socioeconomic status and cultural competence [52]. Despite being a developed economy, Taiwan showed the poorest performance in communication health literacy among the three countries compared. It exhibited the best performance in terms of confusion and functional health literacy. This phenomenon may be attributed to cultural factors in Eastern societies emphasizing obedience and less respect for authority in adolescent ’s decision-making and communication [53, 54]. Item Q7 of the health literacy assessment, which asks whether adolescent feels confused when doctors prescribe medication, had a mean value of 0.64 and a median of 0. A similar pattern was observed for items measuring confusion in health literacy, although there was no floor effect at the item level. While high levels of respect for health professionals and limited emphasis on adolescent ’s autonomy may lead to less skepticism about following medical recommendations, they may also create barriers to communication [55]. Several studies have suggested that children can engage in self-care at an early age [56–58]. However, factors such as a lack of autonomy, time constraints, and other barriers may prevent them from participating in medical decision-making. Empowering adolescent to engage in healthcare and interact with healthcare providers, take responsibility for their health, and address different health-related issues can help improve health literacy.

This study possesses inherent limitations worth noting. The administration of questionnaires at the students’ homes limits our assurance of independent completion. Despite emphasizing the need for genuine and independent responses during classroom instructions, there’s an inherent risk of familial influence on the participants’ responses, potentially introducing bias. It’s essential to weigh this aspect while interpreting our findings. Furthermore, the absence of a gold standard Chinese scale for assessing adolescent health literacy could pose challenges to criterion validity. To address this, we correlated the HAS-A with two health behavior-related scales, the SSB-SE and EES. Additionally, given the sampling constraints, our results might not encompass the health literacy of the entire Taiwanese adolescent demographic. This warrants a cautious approach when drawing comparisons with other global regions. Lastly, a deeper dive into the variables that influence health literacy disparities is required. Our study offers plausible explanations for the observed differences between Taiwan and other countries, but more international research is essential for conclusive insights.

## Conclusion

The study showed that the Chinese version of the HAS-A is a valid and reliable tool for measuring health literacy in late-primary-school-age adolescent with good psychometric properties. The HAS-A scale, particularly in relation to communication health literacy, was consistent with the exploratory nature of the research, as factors such as school grade, parents’ education, and family income were found to be associated with health literacy. The impact of family income on adolescent’s health literacy was more significant than that of the community’s economic status. This study also found disparities in health literacy levels among different countries, with Taiwan having the lowest performance in communication health literacy. This may be owing to cultural factors in Eastern societies emphasizing obedience and respect for authority over adolescent’s decision-making and communication. Therefore, further research is needed to investigate the impact of adolescent’s autonomy on their health literacy and to explore ways to empower them to participate in medical communication and decision-making to improve their health literacy.

### Electronic supplementary material

Below is the link to the electronic supplementary material.

Supplementary Table 1. Percentage of respondents responding to all health literacy items (%)

Supplementary Table 2. Inter-Item Correlations of the Factors Retained

Supplementary Table 3. Results of Confirmatory Factor Analysis for Individual Subscale

## Data Availability

The datasets generated and/or analyzed during the current study are available from the corresponding author on reasonable request.

## List of abbreviations

HAS-A: Health Literacy Assessment Scale for Adolescents
CFA: Confirmatory factor analysis
AGFI: Adjusted goodness-of-fit index
RMSEA: Root mean square error of approximation
CFI: Comparative fit index
NFI: Normed fit index
SSB-SE: Sugar-Sweetened Beverage Self-Efficacy Scale
EES: Emotional Eating Scale
AVE: Average variance extracted
CR: Composite reliability
