# Supplementary material for: Measuring adolescent health literacy in Taiwan: validation of the health literacy assessment scale for adolescents
Source: BMC Public Health. 2023 Dec 4;23:2409. doi: 10.1186/s12889-023-17167-5 (PMC10696858; doi:10.1186/s12889-023-17167-5)
Supplement: Supplementary file 1 — Supplementary Table 1. Percentage of respondents responding to all health literacy items (%) [file 12889_2023_17167_MOESM1_ESM.docx]

**Supplementary Table 1. Percentage of respondents responding to all health literacy items (%)**

|  | Subscale | Items | Never | Rarely | Sometimes | Usually | Always |
| --- | --- | --- | --- | --- | --- | --- | --- |
| Q1 | Communication | How often is it easy for you to ask your doctor questions about your health? | 11.8 | 22.9 | 25.3 | 14.4 | 25.7 |
| Q2 |  | How often does your doctor understand what you mean when you ask them about your health? | 5.9 | 6.9 | 23.3 | 31.5 | 32.4 |
| Q3 |  | How often can you easily describe your health problem to your doctor? | 7.5 | 15.4 | 25.2 | 21.4 | 30.5 |
| Q4 |  | How often does your doctor seem to understand you when you answer a question they ask? | 4.2 | 7.9 | 24.7 | 30.4 | 32.8 |
| Q5 |  | How often do you understand the answers your doctor gives to your questions? | 4.8 | 8.6 | 24.6 | 28.0 | 34 |
| Q6 | Confusion | How often do you need clarification because you find different information about the same health topic? | 29.1 | 26.7 | 24.1 | 11.5 | 8.6 |
| Q7 |  | How often do you get confused when your doctor tells you about taking a medicine? | 63.4 | 20.4 | 8.5 | 3.8 | 3.9 |
| Q8 |  | How often do you need clarification when your doctor tells you about possible side effects from a medicine or treatment? | 40.5 | 28.5 | 18.9 | 6.4 | 5.7 |
| Q9 |  | How often do you need clarification when your doctor tells you about test results, like the results of an X-ray? | 45.2 | 30.4 | 14.5 | 5.0 | 4.9 |
| Q10 | Functional | How often do you need clarification when reading instructions for medicine? | 48.2 | 29.9 | 14.0 | 4.8 | 3.1 |
| Q11 |  | How often do you have problems learning about an illness or health topic because of difficulty understanding the written information you get? | 37.3 | 33.8 | 20.8 | 5.1 | 3.0 |
| Q12 |  | How often do you think the forms you complete at your doctor’s office are confusing? | 53.5 | 26.7 | 13.7 | 3.5 | 2.6 |
| Q13 |  | How often do you need clarification on health information with many numbers and statistics? | 35.8 | 25.5 | 22.4 | 8.4 | 7.8 |
| Q14 |  | When you talk to people other than your doctor about health issues, how often are you trying to understand what they tell you? | 36.8 | 32.5 | 20.5 | 6.2 | 4.0 |
| Q15 |  | When reading brochures or hand-outs about health issues, how often do you need someone to help you read them? | 46.8 | 28.5 | 16.1 | 5.1 | 3.3 |
