# Supplementary material for: Measuring adolescent health literacy in Taiwan: validation of the health literacy assessment scale for adolescents
Source: BMC Public Health. 2023 Dec 4;23:2409. doi: 10.1186/s12889-023-17167-5 (PMC10696858; doi:10.1186/s12889-023-17167-5)
Supplement: Supplementary file 2 — Supplementary Table 2. Inter-Item Correlations of the Factors Retained [file 12889_2023_17167_MOESM2_ESM.docx]

**Supplementary Table 2. Inter-Item Correlations of the Factors Retained**

| The subscale of communication | | | | | | | |
| --- | --- | --- | --- | --- | --- | --- | --- |
|  |  | Q1 | Q2 | Q3 | Q4 | Q5 |  |
| Q1 | Spearman’s rho | - |  |  |  |  |  |
|  | Upper 95% CI | - |  |  |  |  |  |
|  | Lower 95% CI | - |  |  |  |  |  |
| Q2 | Spearman’s rho | 0.453 | - |  |  |  |  |
|  | Upper 95% CI | 0.413 | - |  |  |  |  |
|  | Lower 95% CI | 0.490 | - |  |  |  |  |
| Q3 | Spearman’s rho | 0.497 | 0.584 | - |  |  |  |
|  | Upper 95% CI | 0.459 | 0.552 | - |  |  |  |
|  | Lower 95% CI | 0.530 | 0.615 | - |  |  |  |
| Q4 | Spearman’s rho | 0.392 | 0.635 | 0.597 | - |  |  |
|  | Upper 95% CI | 0.350 | 0.604 | 0.565 | - |  |  |
|  | Lower 95% CI | 0.429 | 0.665 | 0.63 | - |  |  |
| Q5 | Spearman’s rho | 0.358 | 0.464 | 0.477 | 0.562 | - |  |
|  | Upper 95% CI | 0.318 | 0.428 | 0.441 | 0.532 | - |  |
|  | Lower 95% CI | 0.396 | 0.501 | 0.514 | 0.594 | - |  |
| All correlation coefficients are significant (p < .001) | | | | | | |  |
| The subscale of confusion | | | | | | |  |
|  |  | Q6 | Q7 | Q8 | Q9 |  |  |
| Q6 | Spearman’s rho | - |  |  |  |  |  |
|  | Upper 95% CI | - |  |  |  |  |  |
|  | Lower 95% CI | - |  |  |  |  |  |
| Q7 | Spearman’s rho | 0.233 | - |  |  |  |  |
|  | Upper 95% CI | 0.194 | - |  |  |  |  |
|  | Lower 95% CI | 0.272 | - |  |  |  |  |
| Q8 | Spearman’s rho | 0.221 | 0.44 | - |  |  |  |
|  | Upper 95% CI | 0.179 | 0.403 | - |  |  |  |
|  | Lower 95% CI | 0.261 | 0.476 | - |  |  |  |
| Q9 | Spearman’s rho | 0.178 | 0.311 | 0.404 | - |  |  |
|  | Upper 95% CI | 0.136 | 0.273 | 0.364 | - |  |  |
|  | Lower 95% CI | 0.221 | 0.349 | 0.444 | - |  |  |
| All correlation coefficients are significant (p < .001) | | | | | | |  |
| The subscale of functional | | | | | | |  |
|  |  | Q10 | Q11 | Q12 | Q13 | Q14 | Q15 |
| Q10 | Spearman’s rho | - |  |  |  |  |  |
|  | Upper 95% CI | - |  |  |  |  |  |
|  | Lower 95% CI | - |  |  |  |  |  |
| Q11 | Spearman’s rho | 0.477 | - |  |  |  |  |
|  | Upper 95% CI | 0.442 | - |  |  |  |  |
|  | Lower 95% CI | 0.515 | - |  |  |  |  |
| Q12 | Spearman’s rho | 0.382 | 0.402 | - |  |  |  |
|  | Upper 95% CI | 0.343 | 0.363 | - |  |  |  |
|  | Lower 95% CI | 0.419 | 0.438 | - |  |  |  |
| Q13 | Spearman’s rho | 0.384 | 0.426 | 0.406 | - |  |  |
|  | Upper 95% CI | 0.345 | 0.387 | 0.371 | - |  |  |
|  | Lower 95% CI | 0.420 | 0.468 | 0.441 | - |  |  |
| Q14 | Spearman’s rho | 0.363 | 0.413 | 0.383 | 0.435 | - |  |
|  | Upper 95% CI | 0.325 | 0.376 | 0.348 | 0.401 | - |  |
|  | Lower 95% CI | 0.404 | 0.451 | 0.419 | 0.469 | - |  |
| Q15 | Spearman’s rho | 0.263 | 0.314 | 0.282 | 0.305 | 0.300 | - |
|  | Upper 95% CI | 0.222 | 0.276 | 0.241 | 0.268 | 0.262 | - |
|  | Lower 95% CI | 0.306 | 0.353 | 0.322 | 0.345 | 0.340 | - |
| All correlation coefficients are significant (p < .001) | | | | | | | |
