# Supplementary material for: Measuring adolescent health literacy in Taiwan: validation of the health literacy assessment scale for adolescents
Source: BMC Public Health. 2023 Dec 4;23:2409. doi: 10.1186/s12889-023-17167-5 (PMC10696858; doi:10.1186/s12889-023-17167-5)
Supplement: Supplementary file 3 — Supplementary Table 3. Results of Confirmatory Factor Analysis for Individual Subscale [file 12889_2023_17167_MOESM3_ESM.docx]

# Supplementary Table 3. Results of Confirmatory Factor Analysis for Individual Subscales

| Subscale | Item | Factor loading | Composite reliability | Average Variance Extracted |
| --- | --- | --- | --- | --- |
| Communication | Q1 | 0.576 | 0.848 | 0.532 |
|  | Q2 | 0.779 |  |  |
|  | Q3 | 0.763 |  |  |
|  | Q4 | 0.825 |  |  |
|  | Q5 | 0.677 |  |  |
| Confusion | Q6 | 0.327 | 0.632 | 0.313 |
|  | Q7 | 0.570 |  |  |
|  | Q8 | 0.681 |  |  |
|  | Q9 | 0.596 |  |  |
| Functional | Q10 | 0.622 | 0.766 | 0.357 |
|  | Q11 | 0.680 |  |  |
|  | Q12 | 0.611 |  |  |
|  | Q13 | 0.624 |  |  |
|  | Q14 | 0.596 |  |  |
|  | Q15 | 0.421 |  |  |
